# The Development of a Preference for Cocaine over Food Identifies Individual Rats with Addiction-Like Behaviors

**DOI:** 10.1371/journal.pone.0079465

**Published:** 2013-11-18

**Authors:** Adam N. Perry, Christel Westenbroek, Jill B. Becker

**Affiliations:** 1 Molecular and Behavioral Neuroscience Institute, University of Michigan, Ann Arbor, Michigan, United States of America; 2 Department of Psychiatry, University of Michigan, Ann Arbor, Michigan, United States of America; 3 Department of Psychology, University of Michigan, Ann Arbor, Michigan, United States of America; Radboud University, Netherlands

## Abstract

**Rationale:**

Cocaine dependence is characterized by compulsive drug taking that supercedes other recreational, occupational or social pursuits. We hypothesized that rats vulnerable to addiction could be identified within the larger population based on their preference for cocaine over palatable food rewards.

**Objectives:**

To validate the choice self-administration paradigm as a preclinical model of addiction, we examined changes in motivation for cocaine and recidivism to drug seeking in cocaine-preferring and pellet-preferring rats. We also examined behavior in males and females to identify sex differences in this “addicted” phenotype.

**Methods:**

Preferences were identified during self-administration on a fixed-ratio schedule with cocaine-only, pellet-only and choice sessions. Motivation for each reward was probed early and late during self-administration using a progressive-ratio schedule. Reinstatement of cocaine- and pellet-seeking was examined following exposure to their cues and non-contingent delivery of each reward.

**Results:**

Cocaine preferring rats increased their drug intake at the expense of pellets, displayed increased motivation for cocaine, attenuated motivation for pellets and greater cocaine and cue-induced reinstatement of drug seeking. Females were more likely to develop cocaine preferences and recidivism of cocaine- and pellet-seeking was sexually dimorphic.

**Conclusions:**

The choice self-administration paradigm is a valid preclinical model of addiction. The unbiased selection criteria also revealed sex-specific vulnerability factors that could be differentiated from generalized sex differences in behavior, which has implications for the neurobiology of addiction and effective treatments in each sex.

## Introduction

There are 4.1 M people in the USA who have used cocaine in the past year, according to the latest National Survey on Drug Use and Health, among these cocaine users only 1.6 M meet criteria for dependence [1]. When looking at people who have used cocaine at any time approximately 16–17% develop dependence [2]. Therefore, the development of paradigms specifically modeling dependence in laboratory animals is essential for understanding the specific neural substrates of addiction, as opposed to changes resulting from drug use or exposure.

Preclinical addiction models generally attempt to evoke patterns of behavior in rats that mirror those displayed by human addicts [3]–[7]. The diagnostic criteria for dependence in humans are related to changes in an individual’s patterns of consumption and motivation, as reflected in their inability to abstain from use, using larger amounts or for longer periods than intended and giving up important social, occupational or recreational activities due to substance use [8].

Escalation of cocaine intake has been used to model the increased drug consumption characterizing addiction [9]. Animals self-administering cocaine in short access sessions (1–2 hours/day) rapidly stabilize their drug intake, whereas animals self-administering under extended access conditions (typically >6 hours/day) show an escalating pattern of intake over time [10], [11]. Motivation for drugs can also escalate, such that “addicted” rats will expend more effort to continue self-administering cocaine on a progressive ratio schedule [4], [12], [13].

The compulsive nature of addiction not only results in increased consumption and motivation for drugs, but continued use despite adverse consequences or interference with other important activities. Many studies have demonstrated that the concurrent provision of alternative rewards, including food (saccharin/sucrose solutions or pellets) [14], [15] or access to a running wheel [16], can reduce cocaine self-administration. Thus, it has been argued that the minority of rats that choose to self-administer cocaine at the expense of earning alternative rewards represents an “addicted” subpopulation [17].

Finally, addiction in humans is characterized by frequent cycles of abstinence and recidivism of drug use, which has been modeled in laboratory animals with extinction and reinstatement paradigms [18]. Rats with an “addicted” phenotype display greater rates of drug seeking following cocaine priming [3], [5] and exposure to drug cues can reinstate seeking even in the face of adverse consequences [19], [20].

The primary objective of the present study was to examine whether a preference for cocaine over food represents an “addicted” phenotype associated with enhanced motivation for cocaine and greater reinstatement of drug seeking following extinction. Furthermore, we wanted to compare this “addicted” phenotype in males and females, as rates of cocaine dependence are equal or greater in women compared to men [21]–[23] and the neurobiology of cocaine addiction and consequences of cocaine exposure are different in men and women [24]–[27]. Thus, we hypothesized that females would be at increased risk for developing cocaine preferences and show a greater magnitude of addiction-like behaviors than males. Our results confirmed that the development of a cocaine preference was associated with other addiction-like behaviors and demonstrated that females were more likely than males to develop cocaine preferences.

## Methods

### Ethics Statement

All experiments were carried out in accordance with the National Institutes of Health *Guide for the Care and Use of Laboratory Animals* and were preapproved by the University of Michigan Committee on the Use and Care of Animals (PRO00001472, approval date: 11/28/2011). Buprenorphine (0.02 mg/kg, s.c.) was administered for pre-surgical analgesia and surgeries were conducted under isoflurane anesthesia (5% isoflurane in oxygen). Post-surgical pain was managed with additional buprenorphine administered over the following 48 hours as needed.

### Animals

Male and female Sprague Dawley rats were purchased from Charles Rivers (Portage, MI) and were approximately 70–80 days of age upon arrival. Animals were housed in same-sex pairs in standard laboratory cages and maintained on a 14∶10 (light:dark) reversed light cycle (lights off at 08∶00) and provided free access to rat chow and water in a temperature- and humidity-controlled vivarium. At no time were rats subjected to food restriction and a freely accessible water bottle was provided during all self-administration sessions. All experimental procedures were conducted between 09∶00 and 16∶00.

### Catheter Surgeries

Animals were fitted with indwelling jugular catheters constructed from silastic tubing connected to an external guide cannula fixed to a polypropylene mesh [28]. Animals were single-housed after surgery and allowed to recover for at least 5 days before the start of self-administration. Catheters were flushed with 0.2 ml of sterile saline prior to self-administration, which was conducted daily Monday through Friday. Animals were weighed and had their catheters flushed daily with a 0.2 ml solution of gentamicin (3 mg/ml) and heparin (20 U/ml) in bacteriostatic saline. Catheter patency was checked once a week using 0.05–0.1 ml of thiopental sodium (7.5 mg/ml) in sterile saline. Females were vaginally lavaged daily to monitor their estrous cycle.

### Choice Self-administration Paradigm

**Figure 1:**
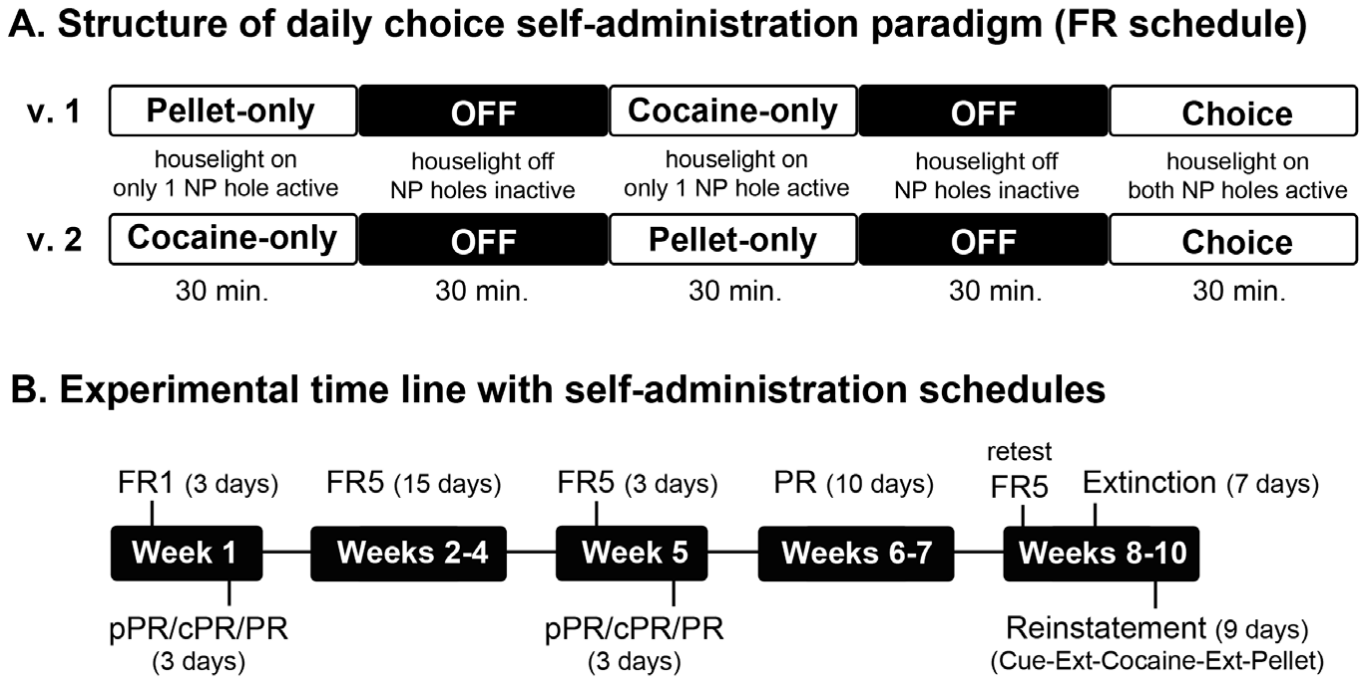
Overview of the choice self-administration paradigm and experimental time line. A. Daily choice self-administration tests are 2.5 hours in duration and consist of 3 active sessions in which reward(s) are available on a fixed ratio (FR) 1 or FR5 schedule with a 40-second time out. The two variants (v.1 and v.2) only differ by the order of the pellet-only and cocaine-only sessions. B. The choice self-administration paradigm (FR1/FR5) was used to identify individual preferences and patterns of intake over time. Motivation was probed early and late in self-administration using a series of progressive ratio (PR) schedules (pPR = pellet-only PR, cPR = cocaine-only PR and PR = concurrent PR). Extinction and reinstatement tests were conducted at the end to examine relapse vulnerability.

Approximately 1–2 hours after lights off, animals were transported to standard operant chambers (Med Associates, Inc., Georgia, VT) and connected to an infusion syringe via a swivel mounted to a counter balanced arm. Subjects were tested in a choice self-administration paradigm consisting of five 30-minute sessions (Fig. 1A). Three of the five sessions were active (pellet-only, cocaine-only and choice sessions), indicated by the illumination of the house light. During active sessions, reward availability (cocaine or pellet) was indicated by the illumination of its respective nose poke cue light. During the pellet-only session, nose pokes in the pellet hole resulted in the delivery of a single food pellet (45 mg banana flavor, BioServ) and transient inactivation of the cue light for 40 seconds. During the cocaine-only session, nose pokes in the cocaine hole resulted in the delivery of a single cocaine infusion (0.4 mg/kg/inf) and transient inactivation of the cue light for 40 seconds. During the choice session, nose poking in either hole resulted in the delivery of its respective reward and inactivation of both holes and their cues for 40 seconds. Nose pokes during the 40-second timeout were recorded but had no programmed consequences. The house light and nose poke cue lights were extinguished during the two intervening “OFF” periods, and nose pokes had no programmed consequences. The order of the cocaine-only and pellet-only sessions was alternated on a daily basis to minimize the effects of perseveration on choice behavior.

Animals were initially trained on a fixed-ratio (FR) 1 schedule (Fig. 1B), in which one nose poke in the active hole was required to trigger reward delivery. After 3 days, motivation for pellets and cocaine was examined in three tests on a progressive-ratio (PR) schedule (see below), after which they were returned to the choice paradigm on an FR5 schedule for an additional 18 days.

Preferences for pellets and cocaine were determined by two complementary methods. First, we directly compared the total number of pellets and infusions earned during each choice session by calculating the percentage of cocaine choices, with >50% cocaine choices indicating a cocaine preference. Second, we standardized the number of pellets and infusions earned during the choice session relative to the respective number of each reward earned in the pellet-only and cocaine-only sessions. This standardization was done because subjects could readily earn the maximum number of pellets in 30 minutes, whereas infusion number was always below the maximum threshold due to the pharmacokinetics of cocaine and the relationship between dose and inter-infusion interval. For the standardized data, a cocaine preference was defined as the maintenance of an infusion ratio that was at least twice as large as the pellet ratio.

By the end of self-administration, both criteria yielded identical preference designations, as most individuals chose one reward to the near complete exclusion of the other. However, as rats first began to develop cocaine preferences, they would often earn a mixture of pellets and infusions in choice sessions making it more difficult to assign a preference based on the percentage of cocaine choices. For example, an individual could receive 20 pellets and 15 infusions in the choice session and appear to have a slight pellet bias (57%), whereas standardization with the data from the pellet-only and cocaine-only sessions (e.g., 40 pellets and 15 infusions, respectively) would indicate a 50% reduction in its pellet intake and a 100% defense of cocaine intake, supporting a strong preference for cocaine. The criteria for cocaine preferences had to be maintained for at least 3 consecutive days in order to be considered a stable preference.

The stability of preferences was determined by examining the percent of FR test days in which the percentage of cocaine choices was >50% (i.e., preference for cocaine), <50% (i.e., preference for pellets) or = 50% (i.e., no preference) over the entire 21 days of FR testing for PP rats, or for the days before and after the designation of cocaine preferences in CP rats. The rationale for these criteria was that if preferences were stable, then PP rats should display a preference for pellets on most test days, whereas CP rats were expected to display a preference for cocaine on most test days once they met the CP criteria.

### Progressive Ratio Schedules

Motivation for cocaine and/or food pellets was tested on a PR schedule in which the response requirement escalated through an exponential series: 1, 3, 6, 9, 12, 17, 24, 32, 42, 56, 73, 95, 124, 161, 208, 268, 346, 445, 573, 737, 948…. adapted from Richardson and Roberts [29]. The final completed response ratio represents the animal’s breaking point (BP). Two different types of PR schedules were utilized in these experiments. First, behavior was examined using pellet-only (pPR) and cocaine-only (cPR) schedules administered on separate days in order to examine the motivation for each reward individually (only one active nose poke hole per day). Motivation for both rewards was then examined simultaneously in a concurrent PR schedule in which both nose poke holes were active on independent PR schedules. The primary purpose of the pPR and cPR schedules was to verify that the self-administration of cocaine did not interfere with the self-administration of the pellet (and vice versa) when both were examined concurrently. All PR tests lasted for 6 hours or until 60 minutes elapsed without earning a reward on either nose poke hole (which ever came first). Completion of the ratio on an active hole resulted in the delivery of its respective reward and inactivation of only that hole for 5 seconds. PR testing was conducted at two time points (Fig. 1B), once early in self-administration after the initial training on the FR1 schedule and again late in self-administration after 18 days on the FR5 schedule.

After the late round of PR testing, all animals continued to self-administer on the concurrent PR schedule for an additional two weeks in order to examine the effects of the estrous cycle on motivation for pellets and cocaine (Fig. 1B). Behavior in females was reported for the following stages: diestrus 1 (D1), diestrus 2 (D2), proestrus/estrus (P/E) and metestrus (M), as the majority of females displayed a 4-day cycle containing a single P, P/E or E lavage. The mean values were used for estrous cycle stages that were repeated during the 10 days of concurrent PR testing.

### Extinction and Reinstatement

All subjects were re-tested once on the FR5 choice paradigm following the PR testing (Fig. 1B). Animals then underwent 7 consecutive days of extinction in which they were placed in the self-administration chambers for 2 hours with the house lights on for the entire session. During extinction, the lights in the nose poke holes were inactivated and there were no consequences to nose poking. After extinction, subjects were tested for reinstatement of cocaine and pellet seeking induced by: 1) reactivation of the light cues associated with each hole, 2) a priming infusion of cocaine, or 3) a priming delivery of a single pellet. Subjects went through an additional three days of extinction training in between each reinstatement test. The nose poke values from the most recent day of extinction were used to analyze behavior in each reinstatement test.

All reinstatement tests were conducted while females were in P/E and one male was yoked to the testing schedule of each female. All reinstatement tests were conducted similar to extinction conditions with the following exceptions. During cue-induced reinstatement, the lights in both nose poke holes were turned on at the start of the session. Nose pokes resulted in the transient inactivation of both cue lights for 40 seconds (similar to the FR1/FR5 choice session), but reward delivery was omitted. During cocaine-induced and pellet-induced reinstatement, animals received either a single priming infusion of cocaine (0.8 mg/kg, i.v.) or one pellet delivered non-contingently at the start of the test. The nose poke cue lights were inactive for the entire duration of the cocaine-induced and pellet-induced reinstatement tests and nose pokes had no consequences.

### Statistics

Statistical analyses were performed with SPSS Statistics (version 20.0). A one-sided z test was used to analyze whether the proportion of females developing cocaine preferences was greater than males. Changes in the proportion of CP individuals over the course of self-administration were examined within each sex by the related samples McNemar test, using the second and eighteenth FR5 tests (days 5 and 21 of total FR testing, respectively). The second FR5 test was used to ensure animals had sufficient self-administration experience prior to assigning a preference and to allow time for their behavior to recover following the transition between the FR1 and FR5 schedules.

All self-administration data were examined for normality via normal Q-Q plots and the Shapiro-Wilk test and homoscedasticity via Levene’s test. The Box-Cox power transformation was applied to identify the optimal transformation for non-normal data [30]. Data from males and females were combined and sex was not considered in the analyses of the FR5 and PR schedules due to the limited number of CP males and the frequent occurrence of non-normal data. Variables that met the assumptions of normality were analyzed with independent samples t-tests to identify the effect of preference. Test statistics and significance values assuming equal variance are reported, unless otherwise indicated by the adjusted degrees of freedom. Mann-Whitney U tests were performed on non-normal data that could not be corrected with transformations. Analyses comparing early and late time points, different test types (e.g., cPR/pPR vs. concurrent PR), or nose poke holes (e.g., pellet hole vs. cocaine hole) were conducted separately within each preference group using the non-parametric Wilcoxon signed rank test for related samples and the original untransformed data.

Analysis of the repeated concurrent PR tests at the end of self-administration was confined to females in order to examine effects of the estrous cycle within each preference group. The data were analyzed by repeated-measures ANOVA with estrous cycle stage as within-subject variable and preference as between-subject variable.

Extinction/reinstatement data were analyzed by repeated-measures ANOVA with test (extinction vs. reinstatement) and nose poke hole (pellet hole vs. cocaine hole) as within-subject variables and preference and sex as between-subject variables. Sex was included as a variable in these analyses as there were more CP males at the time of reinstatement testing and we expected differences between P/E females and males based on other published studies [31], [32].

All repeated-measures ANOVA were conducted with sphericity assumed modeling and Greenhouse-Geisser and Huynh-Feldt adjustments were applied as appropriate [33]. The results of *post hoc* tests were corrected for multiple comparisons using the Bonferonni adjustment and p-values less than 0.05 were considered statistically significant.

## Results

### Development of Cocaine Preferences and Choice Behavior

**Figure 2:**
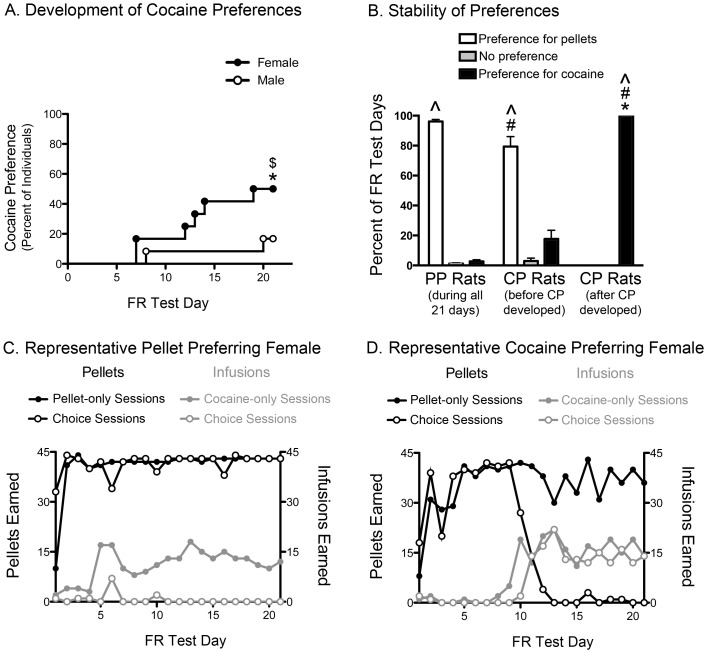
Females are more likely to develop a preference for cocaine. A. The development of cocaine preferences in male (open circles) and female (filled circles) rats (n = 12 per sex). Significant increase in the proportion of cocaine preferring (CP) females between the second and last FR5 tests (* p<0.05). The proportion of CP females was greater than males ($ p<0.05). B. The stability of preferences in PP rats and CP rats (both before and after CP developed). Significant difference between PP and CP rats within same preference category (# p<0.05). Significant difference in preference category before and after CP developed (* p<0.05). Significant difference between the “preference for cocaine” and “preference for pellets” categories within a given group (∧ p<0.05). PP rats (n = 16) and CP rats (n = 8). C. Representative self-administration behavior in a pellet preferring (PP) rat over the 21 FR sessions, displaying the number of infusions (grey) and pellets (black) earned each day during the cocaine-only or pellet-only sessions (closed symbols) and the choice session (open symbols). D. Representative self-administration behavior in a CP rat (CP criteria met on day 11 in this case).

None of the rats met the CP criteria at the beginning of testing (Fig. 2A). By the last FR5 test there was an increase in the proportion of females preferring cocaine (6/12, p = 0.031), but no statistically significant increase in the proportion of CP males (2/12). The proportion of CP females was also greater than that of CP males on the last FR5 test day (p = 0.042). One of the CP males transitioned late (FR test 20) and only had 2 consecutive days of cocaine preferences by day 21. However, given that no other PP rat displayed 2 consecutive days of cocaine preferences (see below), we felt justified in the inclusion of this rat’s data in the CP group.

Preferences were stable during testing for both PP and CP rats (Fig. 2B). PP rats displayed a preference for pellets in 96% of their tests (i.e., 20 out of 21 tests on the FR schedule), which was significantly greater than their percent of days with a cocaine preference (Z = 3.57, p<0.001). The few instances of “no preference” in the PP rats typically occurred during the first few days of testing on the FR1 schedule. In contrast, the few days of “preference for cocaine” in these PP rats tended to occur randomly as isolated “binge” days and never spanned consecutive days, which is distinct from the consistent pattern of cocaine preferences displayed by CP rats after their cocaine preference developed (U = 0.0, n = 24, p<0.001).

Once a rat met the criterion for the CP designation they continued to show a preference for cocaine in 100% of their subsequent tests. This was greater than their percent of days with a preference for pellets (Z = 2.83, p = 0.005), and was significantly different from their percent days with a preference for cocaine before CP developed (Z = 2.53, p = 0.011). Before meeting the CP criteria, these rats had a greater percent of days with pellet preferences than cocaine preferences (Z = 2.37, p = 0.018), which was only slightly lower than that of PP rats (U = 101.0, n = 24, p = 0.023).

Representative profiles of self-administration behavior over the 21 days of FR testing in a pellet preferring (PP) and CP individual are depicted in Figs. 2C and 2D, respectively. As can be seen in these profiles, animals rapidly learned to self-administer the maximum number of pellets, whereas cocaine self-administration emerged more gradually and with greater individual variation. The patterns of nose pokes in each session also demonstrated that animals learned to distinguish reward availability during the active and OFF sessions over time (Table S1).

**Figure 3:**
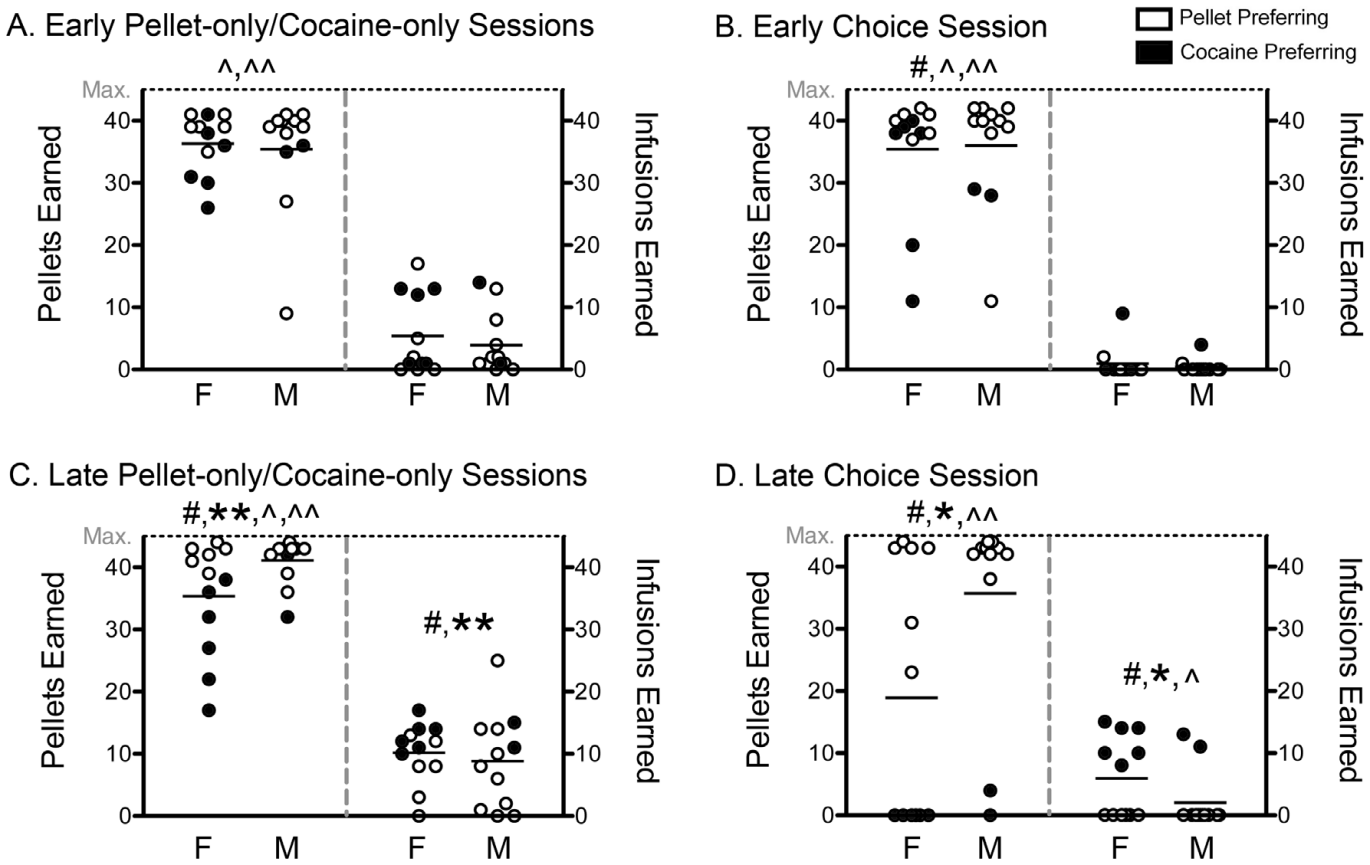
The behavior of cocaine- and pellet-preferring rats diverges after the development of cocaine preferences. Early in self-administration, there are few differences between pellet preferring (PP) and cocaine preferring (CP) rats in the number of rewards earned during the pellet-only and cocaine-only sessions (A) and choice session (B). Late in self-administration, the behavior of PP and CP rats is different in the pellet-only and cocaine-only sessions (C) and non-overlapping in the choice session (D). Each point represents data from a single male (M) or female (F), whereas the horizontal lines represent the means for each sex collapsed across preference groups (PP- white, CP- black). Significant difference between PP and CP rats (# p<0.05), significant difference between early and late time points in PP (** p<0.05) and CP (* p<0.05) rats, and significant difference between the number of pellets and infusions earned within the choice session or between the two single reward sessions in PP (∧∧ p<0.05) and CP (∧ p<0.05) rats. PP females (n = 6), PP males (n = 10), CP females (n = 6) and CP males (n = 2).

The number of infusions earned in the cocaine-only and choice sessions were not significantly different between PP and CP rats in the early FR5 test (Figs. 3A and 3B). PP and CP rats also earned similar numbers of pellets in the early pellet-only session (Fig. 3A), whereas PP earned more pellets in the early choice session (Fig. 3B, U = 107.0, n = 24, p = 0.007). However, PP and CP rats were both choosing pellets over cocaine at this early time point (Z = 3.54, p<0.001; Z = 2.53, p = 0.012, resp.).

Late in self-administration, CP rats earned more infusions in the cocaine-only session (Fig. 3C, t_1,20.55_ = 2.74, p = 0.012) and fewer pellets in the pellet-only session than PP rats (Fig. 3C, t_1,22_ = 5.50, p<0.001). In the late choice session, the numbers of pellets (U = 128.0, n = 24, p<0.001) and infusions (U = 0.0, n = 24, p<0.001) earned by PP and CP rats were completely non-overlapping (Fig. 3D). PP rats exclusively chose pellets over cocaine (Z = 3.55, p<0.001), whereas CP rats chose cocaine over pellets (Z = 2.53, p = 0.012), and these disparities were also reflected in their different percentage of cocaine choices (0% vs. 96.7%, U = 0.0, n = 24, p<0.001).

The differences in choice behavior at the end of self-administration largely reflected changes in CP rats, as they showed a significant increase in infusions (Z = 2.52, p = 0.012) and reduction in pellets (Z = 2.52, p = 0.012) between the early and late tests, which was also reflected in the change in their percentage of cocaine choices over time (5.4% vs. 96.7%, Z = 2.59, p = 0.01).

The behavior of PP rats in the cocaine-only and pellet-only sessions also changed over time, as they earned more infusions (Z = 2.11, p = 0.035) and pellets (Z = 3.11, p = 0.002) late in self-administration, whereas the changes in CP rats over time were not statistically significant. For interested readers, we present these data in the supplementary information analyzed with the PP rats separated into those that robustly self-administered cocaine and those that did not, so called “abstaining” (ABST) rats (refer to Text S1 and Fig. S2).

### Motivation for Cocaine and Natural Rewards

The data obtained under the concurrent PR schedule were comparable to those obtained from the pPR and cPR schedules (Table S2), verifying that motivation for cocaine and food pellets can be measured concurrently on independent PR schedules without interference between the two rewards.

**Figure 4:**
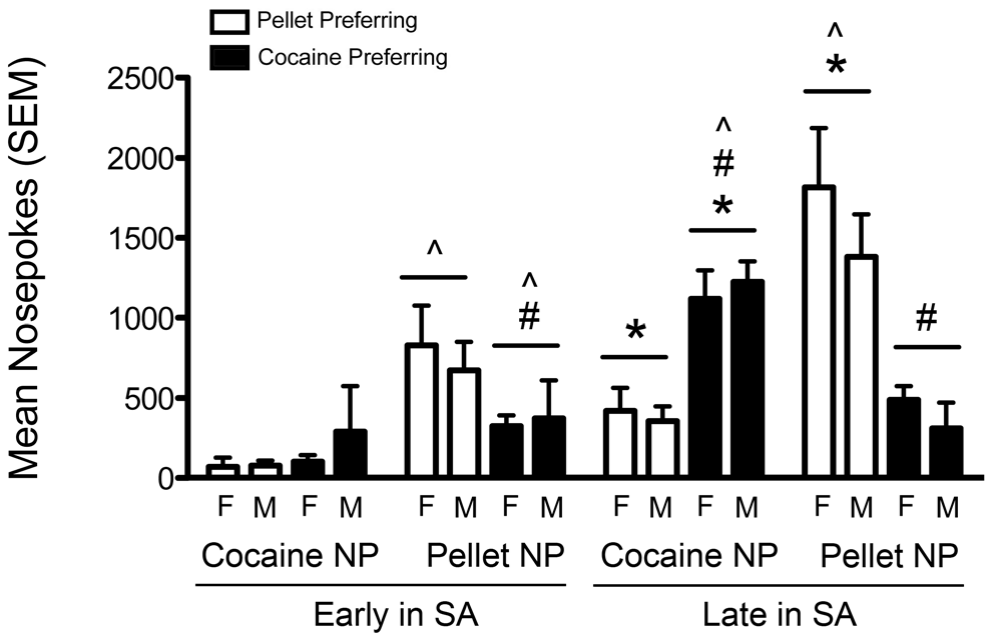
Cocaine preferring rats have increased motivation for cocaine and reduced motivation for pellets. Motivation to self-administer cocaine and pellets in pellet preferring (PP) and cocaine preferring (CP) rats (white and black bars, respectively). Differences between males (M) and females (F) were not examined. Significant difference between PP and CP rats (# p<0.05). Significant difference between early and late in self-administration (* p<0.05). Significant difference between pellet nose pokes (NP) and cocaine nose pokes (NP) within a given group and time (∧ p<0.05). PP females (n = 6), PP males (n = 10), CP females (n = 6) and CP males (n = 2). Vertical lines represent +SEM.

Early in self-administration, there were no significant differences in the motivation for cocaine between PP and CP rats (Fig. 4, see Fig. S1 for data pooled without regard to sex). Both PP rats and those that would go on to become CP individuals were more motivated to work for pellets relative to cocaine (PP- nose pokes: Z = 3.52, p<0.001; BP: Z = 4.20, p<0.001; rewards: Z = 3.53, p<0.001; CP- nose pokes: Z = 2.52, p = 0.012; BP: Z = 2.48, p = 0.013; rewards: Z = 2.38, p = 0.018). However, CP rats demonstrated fewer pellet nose pokes than PP rats (t_1,20.32_ = 2.55, p = 0.019), with similar non-significant trends for pellet BP and pellets earned. By the end of self-administration, CP rats were more motivated to work for cocaine compared to pellets (nose pokes: Z = 2.38, p = 0.017; BP: Z = 2.66, p = 0.008; rewards: Z = 2.40, p = 0.017), whereas PP rats continued to work harder for pellets compared to cocaine (nose pokes: Z = 3.41, p = 0.001; BP: Z = 4.02, p<0.001; rewards: Z = 3.30, p = 0.001). CP rats also displayed greater motivation for cocaine relative to PP rats on all measures (cocaine nose pokes: t_1,22_ = 5.33, p<0.001; cocaine BP: t_1,22_ = 5.09, p<0.001; infusions: t_1,22_ = 4.58, p<0.001), whereas their motivation for pellets was attenuated relative to PP rats (pellet nose pokes: t_1,18.29_ = 4.80, p<0.001; pellet BP: t_1,22_ = 4.92, p<0.001; pellets: t_1,22_ = 4.89, p<0.001).

Both PP and CP rats displayed an increase in motivation for cocaine over time (PP rats- cocaine nose pokes: Z = 2.98, p = 0.003; cocaine BP: Z = 3.11, p = 0.002; infusions: Z = 3.05, p = 0.002; CP rats- cocaine nose pokes: Z = 2.52, p = 0.012; cocaine BP: Z = 2.52, p = 0.012; infusions: Z = 2.53, p = 0.011). Additionally, PP rats displayed an increase in motivation for pellets over time (pellet nose pokes: Z = 3.10, p = 0.002; pellet BP: Z = 3.21, p = 0.001; pellets: Z = 3.28, p = 0.001).

### Effects of the Estrous Cycle on Motivation

**Figure 5:**
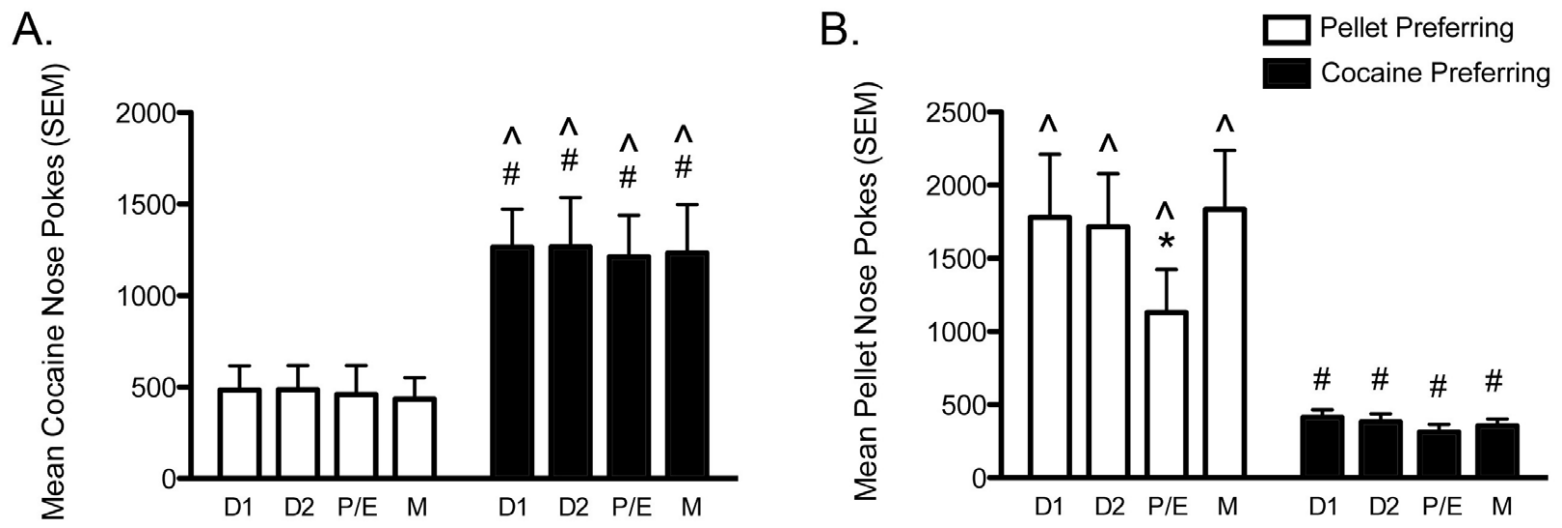
The estrous cycle modulates motivation for pellets, but not cocaine. A. Cocaine preferring (CP) females (black bars) have greater motivation for cocaine than pellet preferring (PP) females (white bars), and neither group displays changes in motivation across the estrous cycle. B. PP females have greater motivation for pellets than CP females during every stage of the cycle. PP females in proestrus/estrus (P/E) have reduced motivation for pellets compared to during diestrus 1 (D1), diestrus 2 (D2) or metestrus (M), whereas there is no effect of the cycle in CP females. (# p<0.05, PP vs. CP; * p<0.05, P/E vs. other stages; ∧ p<0.05, cocaine nose pokes vs. pellet nose pokes). PP females (n = 6) and CP females (n = 6). Vertical lines represent +SEM.

Preference continued to have a strong effect on females’ motivation for cocaine during the two weeks of repeated testing on the concurrent PR schedule (Fig. 5A, Table S3, cocaine nose pokes: F_1,10_ = 8.46, p = 0.016; cocaine BP: F_1,10_ = 8.42, p = 0.016; infusions: F_1,10_ = 7.81, p = 0.019), whereas there were no effects of the estrous cycle and no interactions between estrous cycle stage and preference. CP females displayed more cocaine nose pokes (p<0.026 for all), reached higher cocaine BP (p<0.03 for all) and earned more infusions (p<0.05 for all) during every stage of the cycle compared to PP females.

Preference also continued to affect motivation for pellets (Fig. 5B, Table S3, pellet nose pokes: F_1,10_ = 17.49, p = 0.002; pellet BP:F_1,10_ = 17.57, p = 0.002; pellets: F_1,10_ = 17.09, p = 0.002); however, there was a significant effect of the estrous cycle (pellet nose pokes: F_3,30_ = 11.42, p<0.001; pellet BP: F_3,30_ = 8.91, p<0.001; pellets: F_3,30_ = 10.96, p<0.001) and interaction between preference and estrous cycle stage on the number of pellets earned (F_3,30_ = 3.10, p = 0.042). The simple effects of estrous cycle were also only significant in PP females (pellet nose pokes: F_3,8_ = 11.07, p = 0.003; pellet BP: F_3,8_ = 9.34, p = 0.005; pellets: F_3,8_ = 10.47, p = 0.004), but not CP females. PP females earned fewer pellets (p<0.025 for all), exhibited reduced pellet nose pokes (p<0.013 for all) and reached lower pellet BP (p<0.05 for all) while in P/E compared to every other stage of the cycle. Motivation for pellets did not vary across the estrous cycle in CP females. CP females displayed fewer pellet nose pokes (p<0.008 for all), reached lower pellet BP (p<0.01 for all) and earned fewer pellets (p<0.015 for all) than PP females during every stage of the estrous cycle.

Even though PP females showed a reduction in motivation for pellets during the P/E stage of the estrous cycle, they still displayed more pellet nose pokes than cocaine nose pokes (p<0.002 for all), reached higher pellet BP than cocaine BP (p<0.003 for all) and earned more pellets than infusions (p<0.003 for all) during every stage. Conversely, CP females displayed significantly more cocaine nose pokes than pellet nose pokes (p<0.02 for all), reached higher cocaine BP than pellet BP (p<0.02 for all) and earned more infusions than pellets (p<0.02 for all) during every stage of the estrous cycle.

### Extinction and Reinstatement

An additional CP male was identified during re-testing on the FR5 schedule prior to extinction and this preference shift was further corroborated by changes in cocaine and pellet BP over the 2 weeks of continued PR testing that preceded the FR5 re-test. One PP male was excluded as an outlier from all extinction and reinstatement analyses resulting in the following numbers per group: PP females (n = 6), PP males (n = 8), CP females (n = 6) and CP males (n = 3).

#### Cue-Induced Reinstatement

**Figure 6:**
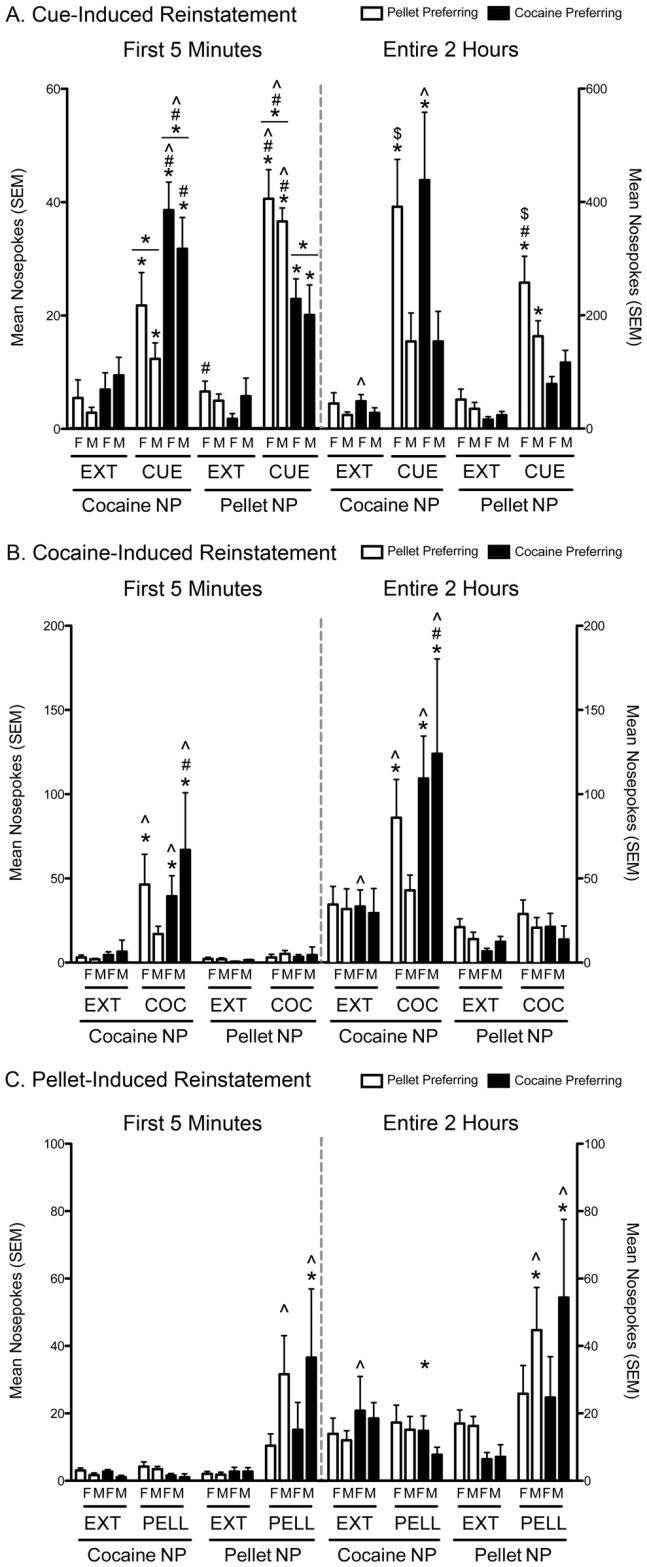
Reinstatement of drug and pellet seeking is influenced by preference and sex. Cocaine seeking and pellet seeking during the first 5 minutes (left side of all figures) and the entire 2 hours (right side of all figures) in male (M) and female (F) pellet preferring (PP) and cocaine preferring (CP) rats (white and black bars, resp.). A. Cue-induced reinstatement (CUE). B. Cocaine-induced reinstatement (COCAINE). C. Pellet-induced reinstatement (PELLET). (* p<0.05, extinction (EXT) vs. reinstatement test; # p<0.05, PP vs. CP; $ p<0.05, M vs. F; ∧ p<0.05, cocaine nose pokes (NP) vs. pellet nose pokes(NP)). PP females (n = 6), PP males (n = 8), CP females (n = 6) and CP males (n = 3). Vertical lines represent +SEM.

The patterns of drug and pellet seeking were different during the first 5 minutes of the last extinction test vs. reinstatement test (Fig. 6A; test: F_1,19_ = 304.88, p<0.001) with the magnitude of poking in the pellet and cocaine holes depending primarily upon preference (prefXhole interaction: F_1,19_ = 26.19, p<0.001; testXprefXhole interaction: F_1,19_ = 14.33, p = 0.001) and sex (testXsex interaction: F_1,19_ = 5.99, p = 0.024). During extinction, CP females displayed fewer pellet nose pokes than PP females (p = 0.036) and poked in the cocaine hole more than pellet hole (p = 0.012). CP and PP males were not significantly different from one another during extinction and did not show a bias for one hole over the other.

All groups showed increased cocaine seeking (p<0.024 for all) and pellet seeking (p<0.022 for all) following reactivation of the cues. However, CP rats poked more in the cocaine hole compared to the pellet hole (p = 0.03) and PP rats poke more in the pellet hole than the cocaine hole (p<0.001). CP rats also displayed more cocaine nose pokes (p = 0.002) and fewer pellet nose pokes (p = 0.001) than PP rats during the reinstatement test.

While nose poking during the first 5 minutes of reinstatement reflected the preference of the animals, a contrasting pattern emerged when examining the data collected over the entire 2-hour test (Fig. 6A). Cue reactivation still increased nose poking (test: F_1,19_ = 79.93, p<0.001); however, the relationship was different for the two nose poke holes (hole: F_1,19_ = 6.30, p = 0.021; holeXtest interaction: F_1,19_ = 7.16, p = 0.015) and largely reflected sex differences in total responding and the effects of preference on pellet seeking (sex: F_1,19_ = 8.08, p = 0.01; sexXhole interaction: F_1,19_ = 5.36, p = 0.032; sexXtest interaction: F_1,19_ = 10.23, p = 0.005; sexXholeXtest interaction: F_1,19_ = 5.31, p 0.033). PP females reinstated cocaine seeking to a higher level than PP males (p = 0.045) and a there was a similar trend in CP rats (p = 0.064). PP females also reinstated pellet seeking to a higher level than PP males (p = 0.035) and CP females (p = 0.001).

#### Cocaine-Induced Reinstatement

The pattern of drug seeking was different during the first 5 minutes of the last extinction test vs. the cocaine-induced reinstatement test (Fig. 6B; test: F_1,19_ = 27.13, p<0.001; hole: F_1,19_ = 27.13, p<0.001; testXhole interaction: F_1,19_ = 30.09, p<0.001) and was influenced by preference and sex (testXholeXprefXsex interaction: F_1,19_ = 4.56, p = 0.046). During the extinction test, all groups showed similar levels of nose pokes for cocaine and pellets and there were no overt biases for one hole over the other, with the exception that CP females continued to display more cocaine than pellet nose pokes (p = 0.001). Cocaine priming significantly increased drug seeking in PP females, CP females and CP males, as all showed an increase in cocaine nose pokes relative to extinction (p<0.02 for all) and directed more pokes toward the cocaine hole than the pellet hole (p<0.015 for all). In contrast, PP males failed to demonstrate significant reinstatement and poked equally in both holes, which also resulted in a significant difference between CP and PP males (p = 0.044). Cocaine priming did not cause reinstatement of pellet seeking.

A similar pattern of results was obtained when examining the data collected over the entire 2 hours of testing (Figure 6B; test: F_1,19_ = 28.26, p<0.001; hole: F_1,19_ = 33.96, p<0.001; holeXpref: F_1,19_ = 5.024, p = 0.034; testXhole: F_1,19_ = 29.05, p<0.001; testXholeXpref: F_1,19_ = 6.87, p = 0.017).

#### Pellet-Induced Reinstatement

The pattern of pellet seeking was different during the first 5 minutes of the last extinction test vs. the pellet-induced reinstatement test (Fig. 6C; test: F_1,19_ = 14.63, p = 0.001; hole: F_1,19_ = 14.25, p = 0.001; testXhole interaction: F_1,19_ = 13.70, p = 0.002) and varied between males and females (holeXsex interaction: F_1,19_ = 4.38, p = 0.050). Again, CP females continued to display more cocaine nose pokes than pellet nose pokes during the extinction test (p = 0.003). Pellet priming significantly induced reinstatement of pellet seeking only in PP and CP males, which both demonstrated increased pellet nose pokes compared to extinction (p<0.035 for both) and poked in the pellet hole more than the cocaine hole (p<0.025 for both). Conversely, the increase in pellet NP between extinction and reinstatement in PP and CP females failed to reach significance and neither group showed a significant bias for the pellet hole following pellet priming.

The same pattern of results were obtained when analyzing the data collected over the entire 2 hours of testing (Fig. 6C; test: F_1,19_ = 12.39, p = 0.002; hole: F_1,19_ = 8.07, p = 0.01; testXhole interaction: F_1,19_ = 17.95, p<0.001; holeXsex: F_1,19_ = 5.06, p = 0.037).

## Discussion

The choice between drugs and alternative rewards has been employed in self-administration studies for decades, largely to understand the behavioral economics of drug reinforcement (reviewed in [34]). Ahmed and colleagues have recently proposed that preferences for cocaine over palatable food rewards can be used to identify vulnerable individuals within the larger population that are relatively resistant to addiction [6], [14], [17], [35]. These studies infer an “addicted” phenotype based on the allocation of choice behavior and the identification of a relatively small proportion of cocaine preferring rats, which fits with the relatively low prevalence of addiction in the human population. Similar to the aforementioned studies and others [36], [37], we also demonstrated that the development of a preference for cocaine over food rewards identified a small group of individuals within the larger population that was able to maintain control over their drug taking. CP rats displayed changes in behavior that capture many features that are critical for the diagnosis of addiction in humans, including an increased frequency of drug taking and forgoing previously enjoyed pursuits [2], [8], [9]. To our knowledge, the present experiment is the first systematic validation that preferences for cocaine over food rewards represent an “addicted” phenotype.

Importantly, the identification of “addicted” (i.e., CP) rats was based on a switch in an individual’s behavior over time, which shares validity with diagnostic criteria used in humans [8]. Choice behavior also circumvents the need to establish arbitrary thresholds to distinguish dependent and non-dependent rats or pin point a more ambiguous transition (e.g., escalation) within a single individual [3]–[5], [10], [12]. Furthermore, the choice paradigm generates two subpopulations that differ in voluntary drug intake when given the same amount of self-administration access. Thus, it is possible to directly compare PP and CP rats without the potential confound introduced by different test conditions [10].

CP rats displayed increased motivation for cocaine on a PR schedule, which is a hallmark of compulsive cocaine intake in preclinical addiction models [3], [4], [11], [13] and distinguishes human addicts from users only meeting abuse criteria [38]. All rats were initially more motivated to self-administer pellets compared to cocaine, which is consistent with the relatively low value of cocaine compared to other rewards [14], [35]. Thus, the different motivational profiles at the end of self-administration represents a true shift by CP rats, as opposed to a pre-existing bias in behavior. The concordance between preferences determined on the FR5 schedule and motivation determined on the PR schedule provides further support that CP rats represent an “addicted” subpopulation that is clearly identifiable even under very different reinforcement schedules.

In addition to motivational changes, CP rats also demonstrated greater recidivism of drug seeking following exposure to cocaine and a drug-paired cue, which further supports our argument that they represent an “addicted” phenotype. Interestingly, responding in the initial five minutes of the cue-induced reinstatement test reliably reflected the preferences of PP and CP rats, whereas their cumulative behavior over the entire two-hour test did not. We propose that the initial response profiles reflect the differential craving of CP and PP rats for their preferred reward, cocaine and pellets respectively, whereas their continued responding over the duration of the test may reflect conditioned responding to the cues. As CP and PP rats self-administered both rewards, albeit to differing degrees, it is not be surprising that they would each display conditioned responses to the pellet and cocaine cues.

The second important finding from the reinstatement tests was the selectivity of reinstatement induced by the primary rewards, as cocaine priming specifically reinstated drug seeking and pellet priming specifically reinstated pellet seeking. Thus, both PP and CP rats appear to detect cocaine’s interoceptive cues, which are then appropriately translated into nose poking in the hole previously associated with cocaine delivery. Tunstall *et al.* [37] showed a similar selectivity for cocaine priming on reinstatement of drug seeking in rats self-administering in a discrete choice procedure, whereas pellet priming induced non-selective seeking of both food and cocaine. The selectivity of pellet priming in our study may reflect methodological differences between studies (e.g., nose poking vs. lever pressing) or proximity of the nose poke hole to the food trough.

### Sex Differences and Effects of the Estrous Cycle

The most pronounced sex difference was in the proportion of individuals developing cocaine preferences, with nearly twice as many females as males displaying this “addicted” phenotype by the end of self-administration. We were unable to statistically compare the discrete behavior of males and females due to the scarcity of CP males; however, there did not appear to be any obvious sex differences under the FR5 or PR schedules. Another preclinical addiction model also failed to identify sex differences in behavior [13], which suggests that while females may be more vulnerable to developing an “addicted” phenotype, its manifestation may be the same in affected males and females.

Interestingly, the various types of reinstatement tests we employed captured the “addicted” phenotype differently in males and females, which is consistent with sex differences in putative drug triggers in human addicts [24], [26], [39]. Cocaine priming reinstated drug seeking equally in CP males and CP females; however, the association between “addicted” phenotype and reinstatement was sexually dimorphic. CP males reinstated drug seeking more than PP males, whereas CP females and PP females displayed equivalent levels of cocaine-induced reinstatement. The disparate relationship between preference and reinstatement of drug seeking may reflect effects of the estrous cycle, as estrous females are generally more sensitive to cocaine-induced reinstatement than males and females in other stages of the cycle [31], [32], [40]. Thus, had we tested non-estrous females, we might have seen the same relationship in both sexes (i.e., greater drug-induced reinstatement in CP rats).

The higher response rates of females during drug cue-induced reinstatement are consistent with other types of reinstatement studies [41]–[43], which have generally been interpreted to reflect greater craving and relapse vulnerability in females. However, as CP males and females had equivalent cocaine nose pokes during the first five minutes of testing, we argue that both sexes experience equal craving following initial exposure to the drug cue, similar to reports in human addicts [26], [44]. The sex difference that emerges when the entire two-hour test is considered may therefore reflect other processes distinct from craving, such as conditioned responding to the drug-paired cue or sex differences in cognitive and behavioral strategy. Our data further suggest that this sex difference may not relate to differential vulnerability to addiction, as the magnitude of seeking was similar in PP and CP females.

CP females also displayed an attenuated cue-induced reinstatement of pellet seeking relative to PP females, which is consistent with their lesser motivation to work for pellets on the PR schedule and may reflect active devaluation of the cue associated with the pellet. The examination of cue-induced reinstatement before and after the development of cocaine preferences would clarify whether this reinstatement deficit in CP females reflects active devaluation of the pellet and its cue, or a pre-existing group difference. Neither female group reinstated pellet seeking following non-contingent pellet delivery. We propose that this also reflects devaluation of the natural reward in CP females, whereas it may primarily reflect estrous cycle effects on motivation for food in PP females. Future studies examining pellet-induced reinstatement in other stages of the cycle in PP and CP females will be required to clarify the contributions of preference and the estrous cycle.

CP males appeared to respond to the pellet and its cue in a similar fashion as their PP counterparts, suggesting that CP males may not experience the same level of reward devaluation as CP females. Alternatively, males may recover their interest in natural rewards and their cues more quickly during abstinence than females. Appetitive responses for food and other rewards are blunted for several days following amphetamine withdrawal in male rats [45]–[47]; however, to our knowledge sex differences in appetitive responses following withdrawal from cocaine self-administration have not been reported. Either way, it is clear that the “addicted” phenotype manifests differently in males and females in regards to reinstatement behavior directed towards natural rewards and their cues, which may relate to our novel findings of estrous cycle effects on motivation for food rewards.

There was a clear effect of reproductive state on motivational processes in females, even though individual preferences were stable over the estrous cycle, consistent with the results of another discrete choice procedure [36]. We anticipated that motivation for cocaine would vary over the estrous cycle and were surprised to identify estrous effects on motivation for food, but not cocaine. However, the reports of estrous cycle effects on self-administration behavior are mixed.

In general, motivation for drugs is increased in P/E females relative to other stages, whereas motivation for food has been reported not to vary much across the estrous cycle [48]–[51]. Our novel finding of estrous cycle effects on motivation for food (but not cocaine) is consistent with the reduced food intake and pronounced weight loss displayed by female rodents in this stage [52]–[55]. Importantly, even though PP females displayed reduced motivation for food during the P/E stage of the cycle, they continued to maintain a higher BP for food relative to cocaine, which may explain why their preferences were stable across the estrous cycle.

The unchanging motivation for cocaine across the estrous cycle may reflect the methodological differences with other studies discussed above (e.g., FR schedule, cocaine dose, presence of an alternative reward and testing during the dark phase of the light cycle). However, it is also consistent with several other studies in rodents and primates that demonstrate no effect of reproductive cycle or gonadal hormones on cocaine self-administration [56]–[59].

### Considerations and Caveats

Our choice self-administration paradigm has several unique features that distinguish it from others. First is the inclusion of choice sessions from the first day of testing. Many other paradigms involve a training phase in which cocaine and the non-drug reward are self-administered separately on alternating days before choice sessions are instituted [35]–[37]. Therefore, by the time preferences are assessed it is possible that many or all of the vulnerable rats will already display cocaine preferences. Not only does this preclude analyzing group differences in the rates at which cocaine preferences develop, but it also makes it difficult to study potential factors that might contribute to their development.

We did not explicitly compare the rates of CP formation in males and females, largely due to the small number of CP males. However, based on the relative timing that cocaine preferences emerged in each sex, it would seem that while females are more vulnerable to developing cocaine preferences, they do not necessarily progress earlier than males, how this might relate to the “telescoping” effect described in clinical reports is unclear [60]. It would be interesting to model both the rate and frequency of cocaine preference development in future studies, particularly when examining groups in which the rate parameter might be a more sensitive index of vulnerability (e.g., stressed vs. non-stressed individuals).

The gradual increase in CP rats that we observe over time likely reflects the interaction between vulnerability factors and self-administration experience that are unique to each individual. The final proportions of CP rats that we obtained (∼25% in males and ∼50% in females) are generally similar to those reported by others using different methodologies and varying lengths of self-administration [35]–[37]. However, it is uncertain whether these proportions would remain stable or continue to increase with greater lengths of self-administration. Cantin *et al.* [35] have shown that the proportion of CP individuals is unrelated to an individual’s prior history of cocaine intake, which is also consistent with what we have seen, and suggests that there is not a fixed “tipping point” of intake after which one becomes “addicted.” However, in other preclinical dependence models, longer periods of self-administration (either in terms of total days or hours per day) have been suggested to increase vulnerability [5], [10], [13], [61].

A second strength of our model is the continued testing of animals with the cocaine-only and pellet-only sessions. In other choice paradigms the sessions in which only a single reward is available are often omitted once animals have completed their training phase [36], [37]. Thus, here it is possible to monitor changes in the consumption of each reward when the other is not present. In other paradigms, the number of trials in which only a single reward is available is kept relatively limited [35], which may not be ideal for demonstrating loss of interest in the natural reward, particularly as it seems to be highly variable amongst individuals in our study (ranging from ∼ 0–50% suppression).

While the difference in pellet intake between CP and PP rats during the pellet-only sessions may seem modest, we propose that it is highly relevant to DSM criteria that are often neglected in preclinical models, namely the loss of interest in previously enjoyed activities (e.g., recreation, work, social interactions, *et cetera*). Our data show that not only do CP rats earn fewer pellets, suggesting a loss of interest in this natural reward over time, but that CP rats are also less motivated to work for pellets (males and females) and are less responsive to their conditioned cues (only females) than PP rats. While these deficits in motivation and reinstatement behavior may not directly demonstrate a loss of interest in the previously enjoyed pellets, they do provide circumstantial evidence that “addicted” individuals do not respond as vigorously to the pellet and its cue.

CP rats did we not exhibit a reduction in their pellet BP between the early and late PR tests. One might argue that this does not reflect a loss of previously enjoyed reinforcers. These tests of motivation were conducted when animals had very different histories of self-administration training (3 days on a FR1 schedule vs. weeks on a FR5 schedule). In this light, the increased pellet BP over time displayed by PP rats likely reflects the natural trajectory for PR responding as animals gain greater self-administration experience. So the fact that the CP rats do not show a similar increase in pellet BP over time indicates at least that the CP rats are less motivated to receive pellets than PP rats. As most CP rats robustly self-administered pellets before developing cocaine preferences and continued self-administering pellets (albeit much fewer) afterward, it seems unlikely that their PR responding would fail to be enhanced by training. We suggest that the motivation for pellets actually diminishes as CP rats develop cocaine preferences, which would also be consistent with their reduced pellet intake in the pellet-only sessions. Thus, had we examined PR responding more frequently, we would have expected to see pellet BPs wax and wane, as the initially pellet-preferring CP rats gained self-administration experience and then transitioned to their final CP status.

In addition to its role in identifying the loss of interest in the previously enjoyed pellets, we also found continued tracking of pellet and cocaine intake during single reward sessions to be useful for standardizing choice behavior. In our study, all animals displayed very polarized preferences, such that the standardized choice data yielded identical preference designations as more straightforward criteria (e.g., >50% cocaine choice = cocaine preference). However, if the preferences of animals are more ambiguous and closer to 50%, then the standardized criteria may resolve preferences more reliably by determining which reward is “better defended” (i.e., intake in the choice session more closely matches intake in the absence of an alternative). Such standardized criteria might also disambiguate whether a cocaine preference truly reflects a vulnerable individual, or merely an individual responding to the behavioral economics of choosing between a given dose of cocaine and an alternative reward. This could be especially important when high cocaine doses are used, as small shifts in infusion and pellet number can dramatically alter the percent cocaine choice and inadvertently label an individual as cocaine preferring even if they are not truly “addicted.”

The dose of cocaine utilized in choice paradigms deserves special consideration, as there are several examples in which cocaine preferences become more robust at higher unit doses [36], [62], [63]. However, other studies have shown that high doses of cocaine are not necessarily preferred over food when longer inter-trial intervals are imposed [14], [36]. Therefore, the acute effects of cocaine may bias choice behavior towards cocaine preferences even if food is still more highly valued. Thus, the use of low to moderate doses of cocaine and/or longer post-infusion time out periods is warranted to ensure that cocaine preferences can be used as reliable indicators of the “revaluation” of cocaine over food in vulnerable individuals, as opposed to just reflecting the acute intoxicating effects of cocaine on choice behavior.

### Summary and Conclusion

In conclusion, we have validated that the development of a preference for cocaine over a food reward represents an “addicted” phenotype that is associated with increased frequency of drug taking, enhanced motivation for drugs, increased relapse potential and a loss of interest in non-drug rewards. Understanding the neurobiology contributing to this loss of interest may provide new treatment avenues for addiction, as reinvigorating enjoyment of natural rewards may help individuals shift their behavior away from using drugs and prevent relapse. Furthermore, the choice self-administration paradigm appears ideally suited for examining sex differences in cocaine dependence, as the same unbiased criteria can be used to identify “addicted” males and females, unlike many other measures that can be affected by sex differences in response rates and behavioral strategies. The development of valid preclinical models that capture these and other sex differences in behavior are essential for clarifying the neurobiology of addiction in males and females, which could lead to the development of novel pharmacotherapies tailored to the specific needs of men and women.

## Supporting Information

Figure S1 **Cocaine preferring rats have increased motivation for cocaine and reduced motivation for pellets.** Same data and analyses as in Fig. 4, but depicted without regard to sex. Significant difference between PP and CP rats (# p<0.05). Significant difference between early and late in self-administration (* p<0.05). Significant difference between pellet nose pokes (NP) and cocaine nose pokes (NP) within a given group and time (∧ p<0.05). PP rats (n = 16) and CP rats (n = 8). Vertical lines represent +SEM.
(TIF)

Figure S2 **Cocaine preferring rats have increased motivation for cocaine and reduced motivation for pellets.** Preference groups with different letters are significantly different from one another (p<0.05). Significant difference between early and late in self-administration (* p<0.05). Significant difference between pellet nose pokes (NP) and cocaine nose pokes (NP) within a given group and time (∧ p<0.05). ABST rats (n = 6), PP rats (n = 10) and CP rats (n = 8). Vertical lines represent +SEM.
(TIF)

Table S1 **Summary of nose poke data from active (cocaine-only, pellet-only and choice) and inactive (“OFF”) sessions during the early and late FR5 tests.**
(TIF)

Table S2 **Comparison of data from the single and concurrent reward schedules during the early and late PR tests.**
(TIF)

Table S3 **Effects of the estrous cycle on motivation parameters (nose pokes, BP and rewards) during the late repeated concurrent PR tests.**
(TIF)

Text S1 **Additional analyses and discussion of FR5 and PR data with PP rats separated into two subgroups, those that readily self-administered cocaine during cocaine-only sessions and those that largely abstained from cocaine self-administration.**
(DOCX)
