# Supplementary figures and images for: The Development of a Preference for Cocaine over Food Identifies Individual Rats with Addiction-Like Behaviors
Source: PLoS One. 2013 Nov 18;8(11):e79465. doi: 10.1371/journal.pone.0079465 (PMC3832528; doi:10.1371/journal.pone.0079465)

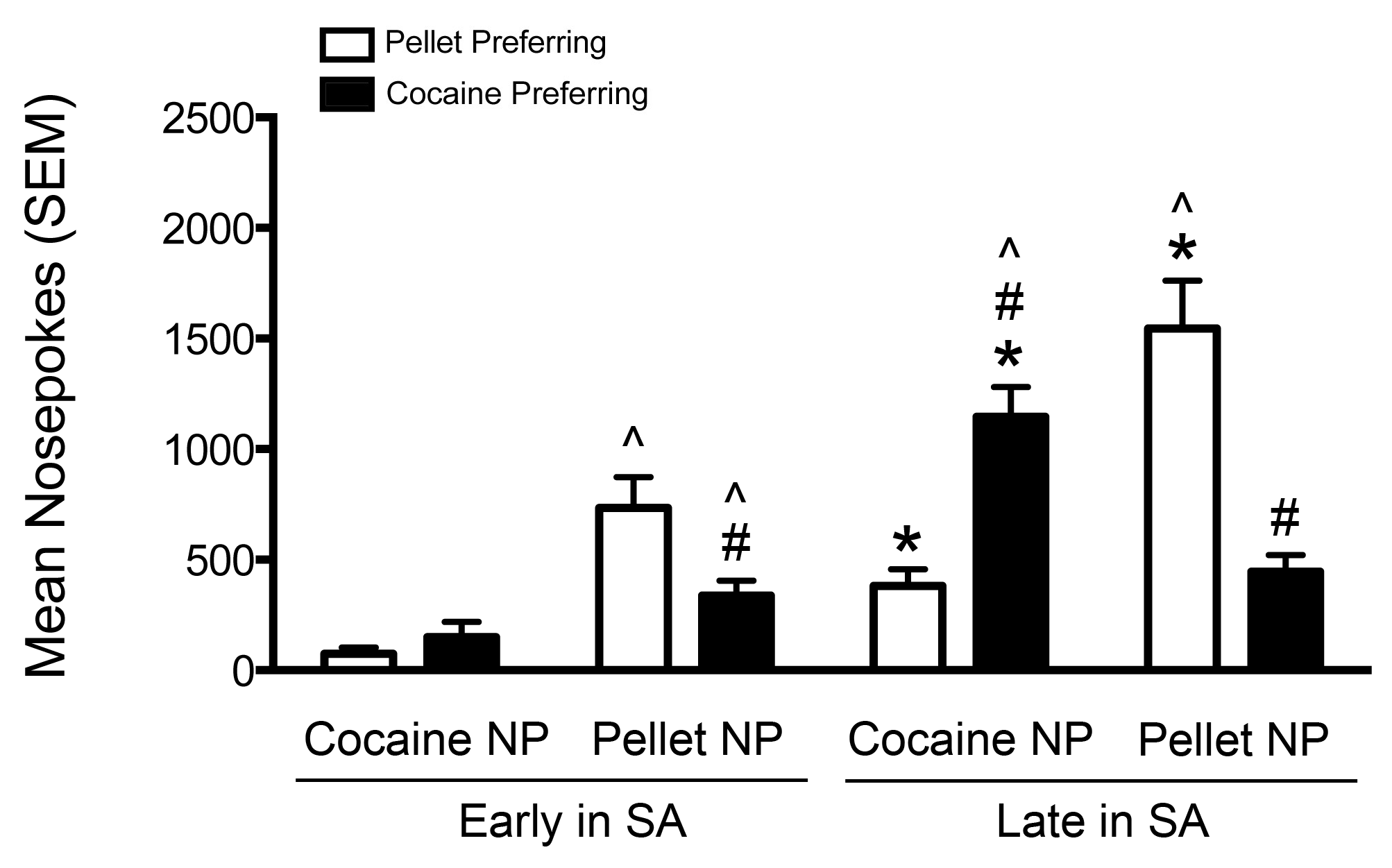

Supplement: Figure S1 — Cocaine preferring rats have increased motivation for cocaine and reduced motivation for pellets. Same data and analyses as in Fig. 4, but depicted without regard to sex. Significant difference between PP and CP rats (# p<0.05). Significant difference between early and late in self-administration (* p<0.05). Significant difference between pellet nose pokes (NP) and cocaine nose pokes (NP) within a given group and time (∧ p<0.05). PP rats (n = 16) and CP rats (n = 8). Vertical lines represent +SEM. (TIF) [file pone.0079465.s001.tif]

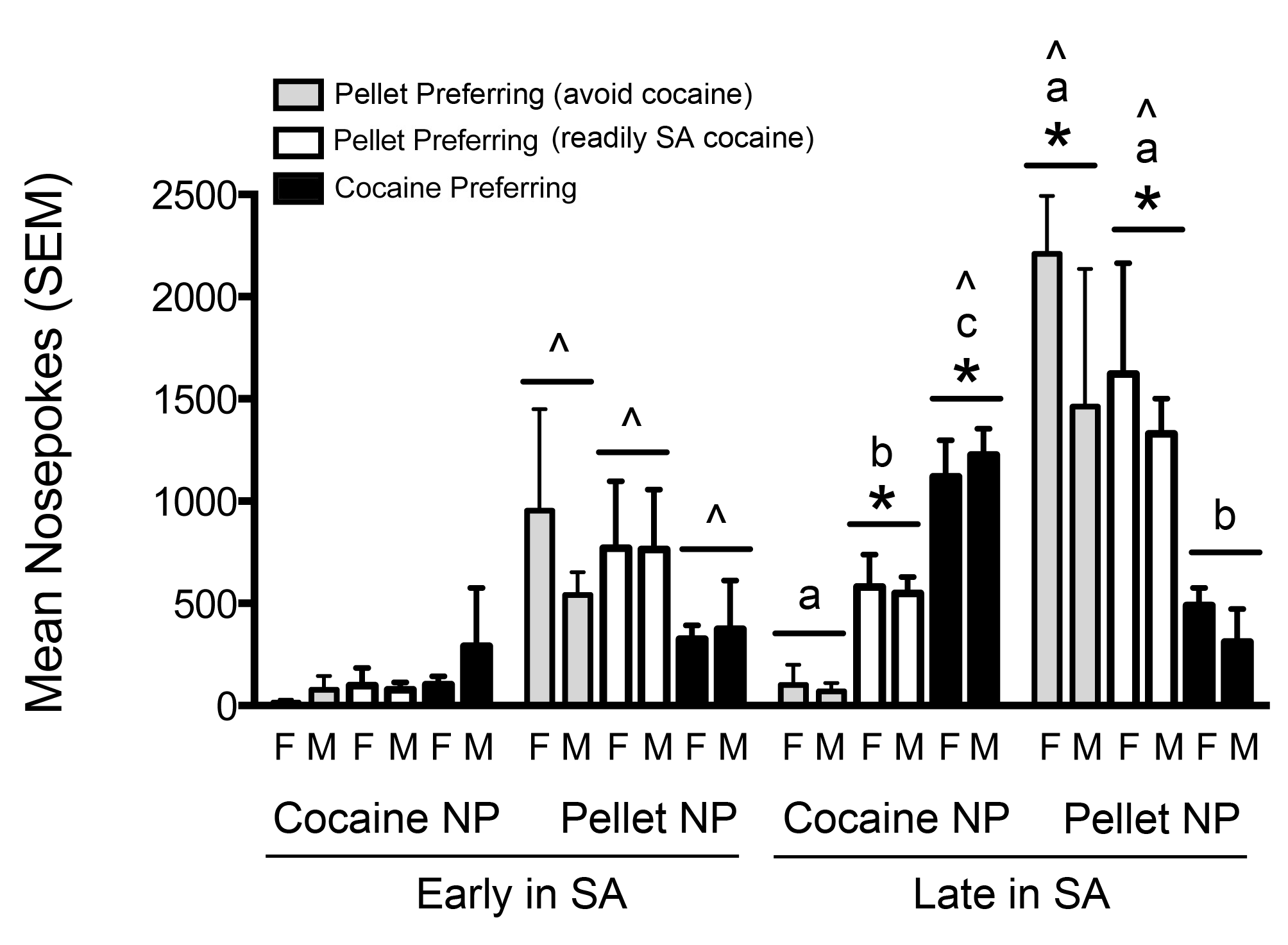

Supplement: Figure S2 — Cocaine preferring rats have increased motivation for cocaine and reduced motivation for pellets. Preference groups with different letters are significantly different from one another (p<0.05). Significant difference between early and late in self-administration (* p<0.05). Significant difference between pellet nose pokes (NP) and cocaine nose pokes (NP) within a given group and time (∧ p<0.05). ABST rats (n = 6), PP rats (n = 10) and CP rats (n = 8). Vertical lines represent +SEM. (TIF) [file pone.0079465.s002.tif]

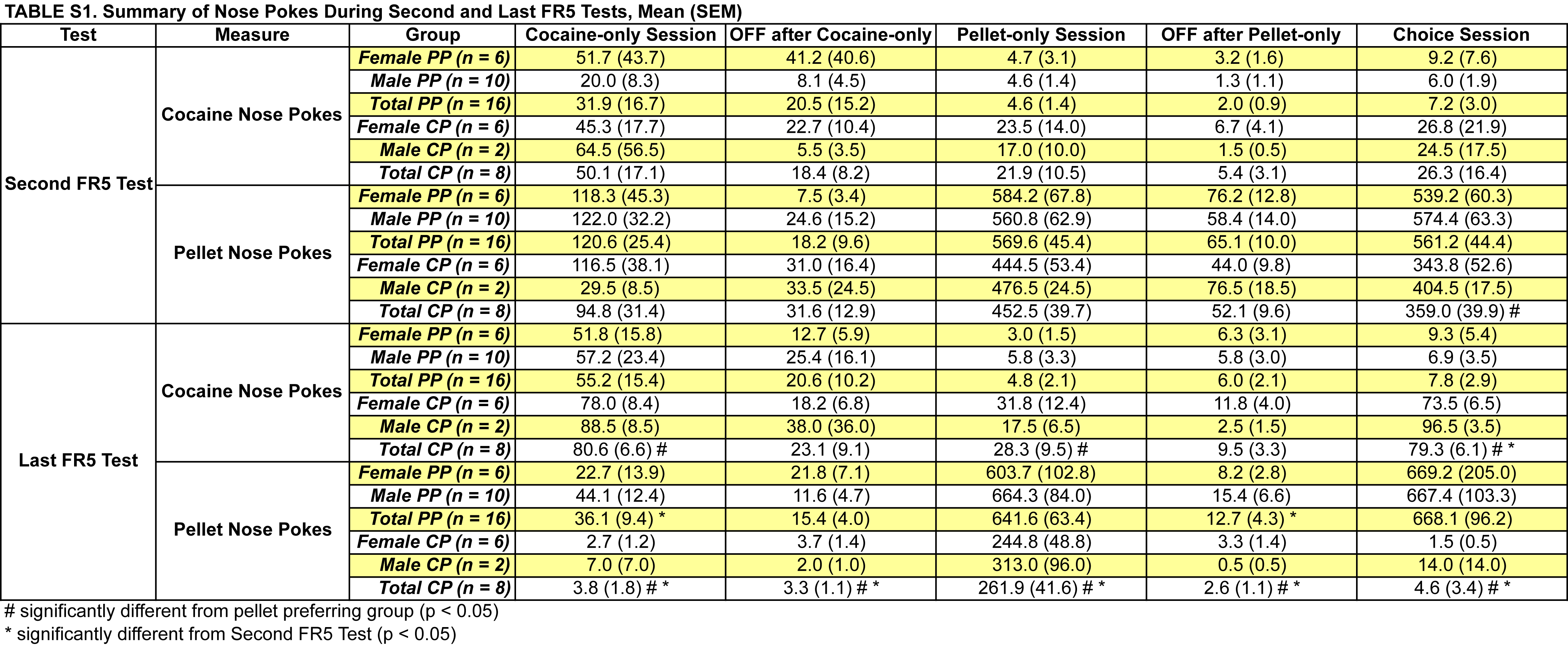

Supplement: Table S1 — Summary of nose poke data from active (cocaine-only, pellet-only and choice) and inactive (“OFF”) sessions during the early and late FR5 tests. (TIF) [file pone.0079465.s003.tif]

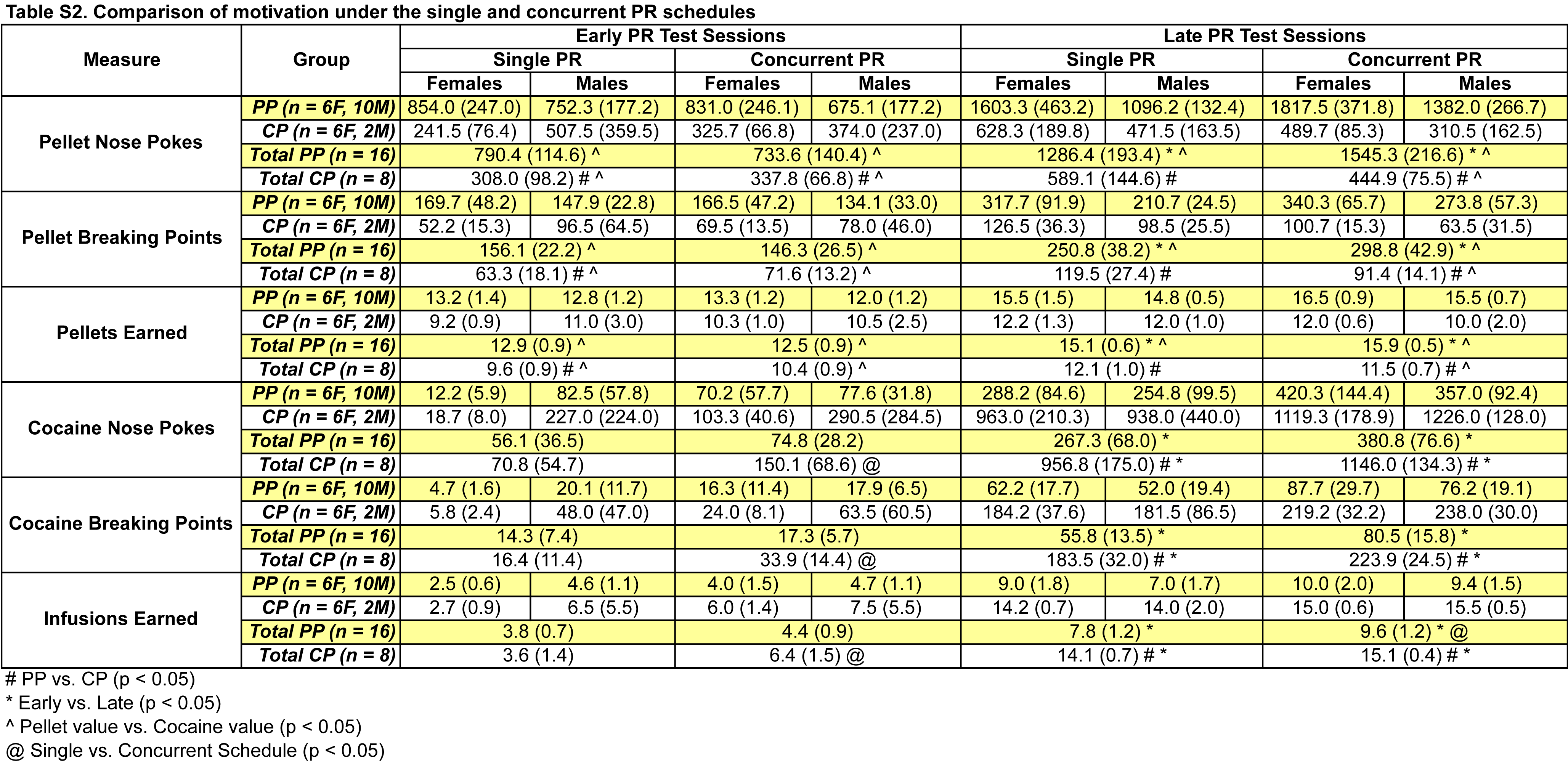

Supplement: Table S2 — Comparison of data from the single and concurrent reward schedules during the early and late PR tests. (TIF) [file pone.0079465.s004.tif]

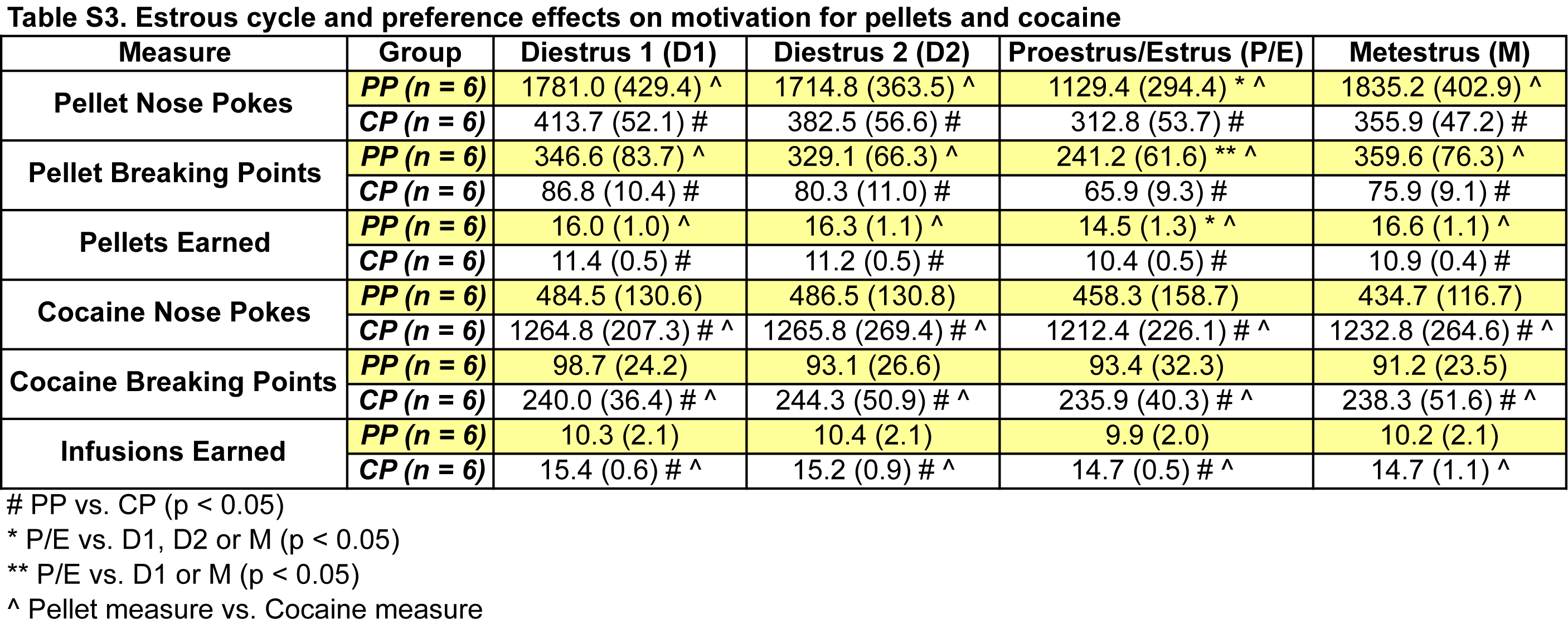

Supplement: Table S3 — Effects of the estrous cycle on motivation parameters (nose pokes, BP and rewards) during the late repeated concurrent PR tests. (TIF) [file pone.0079465.s005.tif]
