# Supplementary material for: The Development of a Preference for Cocaine over Food Identifies Individual Rats with Addiction-Like Behaviors
Source: PLoS One. 2013 Nov 18;8(11):e79465. doi: 10.1371/journal.pone.0079465 (PMC3832528; doi:10.1371/journal.pone.0079465)
Supplement: Text S1 — Additional analyses and discussion of FR5 and PR data with PP rats separated into two subgroups, those that readily self-administered cocaine during cocaine-only sessions and those that largely abstained from cocaine self-administration. (DOCX) [file pone.0079465.s006.docx]

**Supplementary Statistics**

For analyses in which PP rats were separated into those that abstained from cocaine self-administration (ABST) and those that readily self-administered cocaine (PP), variables meeting the assumptions for parametric statistics were analyzed with ANOVA with preference (ABST, PP or CP) as the between-subject variable. Non-normal data were analyzed with the independent-samples Kruskal-Wallis test.

**Supplementary Results**

We reanalyzed the data to determine whether the differences between PP and CP rats were influenced by the subgroup of abstaining (ABST) rats within the PP population. The reanalysis demonstrated a significant effect of preference on infusions in the late cocaine-only session (H= 13.97, d.f.= 2, p= 0.001), with ABST rats self-administering fewer infusions than both PP rats (p= 0.01) and CP rats (p= 0.001) and no significant difference between PP and CP rats. There was a significant effect of preference on the number of pellets earned in the late pellet-only session (H= 13.05, d.f.= 2, p= 0.001), with CP rats earning fewer pellets than both PP rats (p= 0.005) and ABST rats (p= 0.006). All other analyses showed the same pattern of results as when the data from the total PP population was compared to the CP rats, suggesting that the only real difference between ABST and PP rats was in the amount of cocaine self-administered in the cocaine-only sessions.

We found the same pattern of results for the PR tests when the ABST rats were separated into their own group for analysis (Fig. S2). During the early PR test, all groups nose poked in the pellet hole more than the cocaine hole (ABST: Z= 2.20, p= 0.028; PP: Z= 2.80, p= 0.005; CP: Z= 2.52, p= 0.012). During the late PR test, CP rats switched to nose poking more in the cocaine hole than the pellet hole (Z= 2.38, p= 0.017), whereas ABST and PP rats continued to nose poke more in the pellet hole (Z= 2.20, p= 0.028; Z= 2.60, p= 0.009, resp.). There was no effect of preference on pellet or cocaine nose pokes early in self-administration, whereas preference had a significant effect on both cocaine and pellet nose pokes late in self-administration (F_2,21_= 27.06, p< 0.001; F_2,21_= 6.24, p= 0.007, resp.). The number of cocaine nose pokes differed between all 3 groups (CP > PP > ABST, p< 0.01 for each comparison), whereas only CP rats differed in terms of pellet nose pokes (p< 0.03 for each comparison). Only PP and CP rats showed an increase in cocaine nose pokes between the early and late tests (Z= 2.80, p= 0.005; Z= 2.52, p= 0.012, resp.), whereas only ABST and PP rats showed an increase in pellet nose pokes between the two tests (Z= 2.20, p= 0.028; Z= 2.29, p= 0.022, resp.).

**Supplementary Discussion**

An additional novel finding from this study was the presence of a subgroup of PP rats that did not robustly self-administer cocaine even when it was the only reward available. While it is difficult to rule out that these “abstaining” individuals just never learned to self-administer cocaine, they readily acquired pellet self-administration, so there was no learning deficit in these individuals. Many of these ABST rats continued to earn approximately 1 or 2 infusions a day, suggesting that they were aware of the cocaine hole.

The low number of infusions self-administered by these ABST rats may have been insufficient to reinforce robust self-administration behavior, or having access to an alternative reward may have reduced their drive to self-administer cocaine, similar to the effects of environmental enrichment and discrete access to sweets [1-3]. It is also possible that the ABST rats find the aversive effects of cocaine more overwhelming than other PP rats, which could cause them to avoid cocaine and appear to not acquire drug self-administration [4]. It is possible that these aversive effects might have an even greater effect on self-administration behavior when they can be directly contrasted with the appetitive effects of the natural reward, such as in our choice paradigm.

**Supplementary References**

1. Liu C, Grigson PS (2005) Brief access to sweets protect against relapse to cocaine-seeking. Brain Res 1049: 128–131. doi:10.1016/j.brainres.2005.05.013.

2. Puhl MD, Blum JS, Acosta-Torres S, Grigson PS (2012) Environmental enrichment protects against the acquisition of cocaine self-administration in adult male rats, but does not eliminate avoidance of a drug-associated saccharin cue. Behav Pharmacol 23: 43–53. doi:10.1097/FBP.0b013e32834eb060.

3. Cason AM, Grigson PS (2013) Prior access to a sweet is more protective against cocaine self-administration in female rats than in male rats. Physiol Behav 112-113: 96–103. doi:10.1016/j.physbeh.2013.02.017.

4. Rademacher DJ, Anders KA, Thompson KJ, Steinpreis RE (2000) The failure of some rats to acquire intravenous cocaine self-administration is attributable to conditioned place aversion. Behav Brain Res 117: 13–19.
